# Supplementary material for: Metabolomic Profiling and Cytotoxic Tetrahydrofurofuran Lignans Investigations from Premna odorata Blanco
Source: Metabolites. 2019 Oct 13;9(10):223. doi: 10.3390/metabo9100223 (PMC6836009; doi:10.3390/metabo9100223)
Supplement: Supplementary file 1 [file metabolites-09-00223-s001.pdf]

Supplementary Information

Table of Contents

Figure legends | Page No.

Figure legends	Page No.
**Figure S1.** ^1H NMR spectrum of compound **35** measured in $CDCl_3$ at 400 MHz.	**S5**
**Figure S2.** DEPT-Q NMR spectrum of compound **35** measured in $CDCl_3$ at 100 MHz.	**S6**
**Figure S3.** HSQC spectrum of compound **35** measured in $CDCl_3$.	**S7**
**Figure S4.** HMBC spectrum of compound **35** measured in $CDCl_3$.	**S8**
**Figure S5.** HRESIMS spectrum of compound **35**.	**S9**

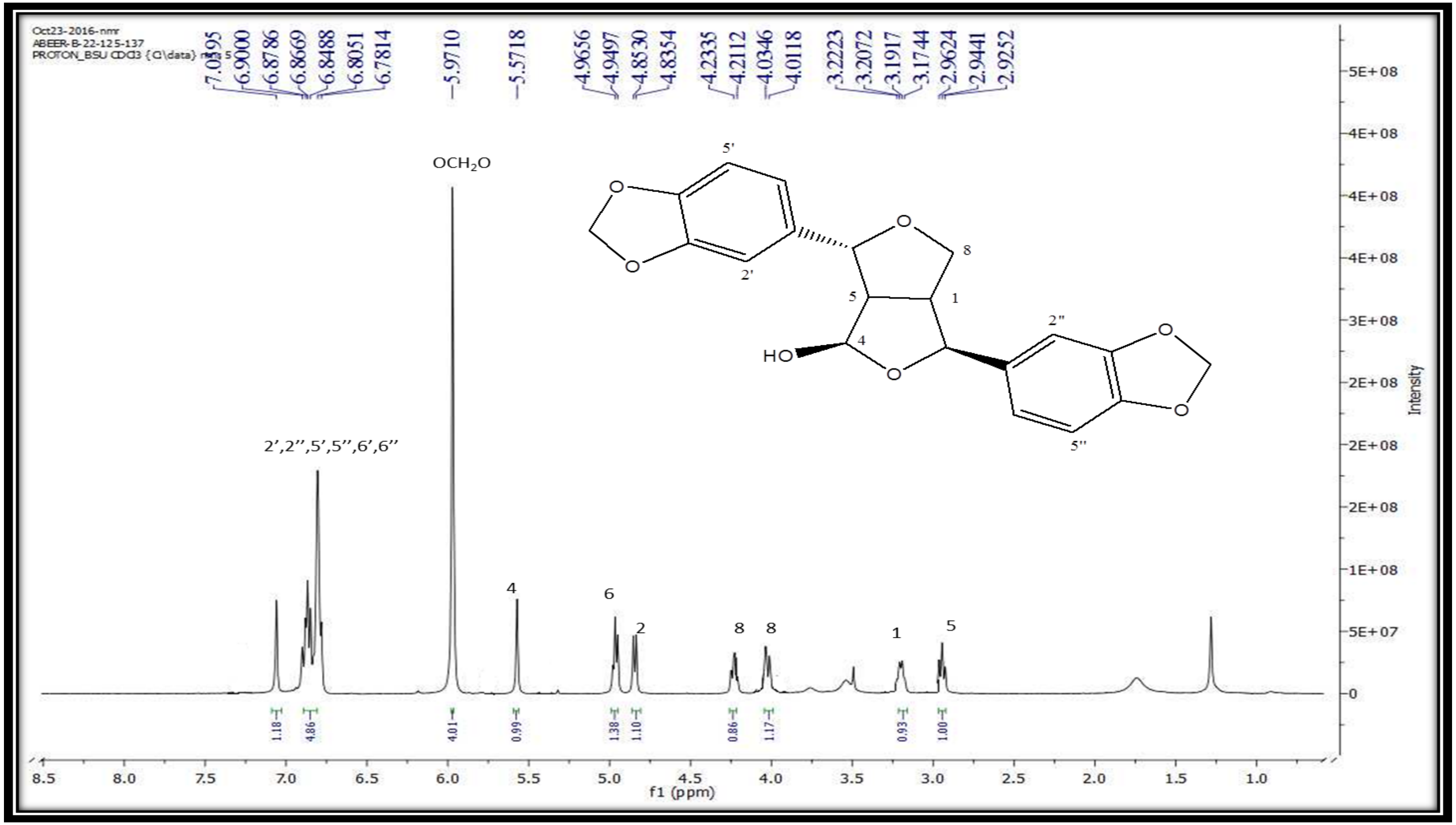

Figure S1. ^{1}H NMR spectrum of compound **35** measured in $CDCl_3$ at 400 MHz.

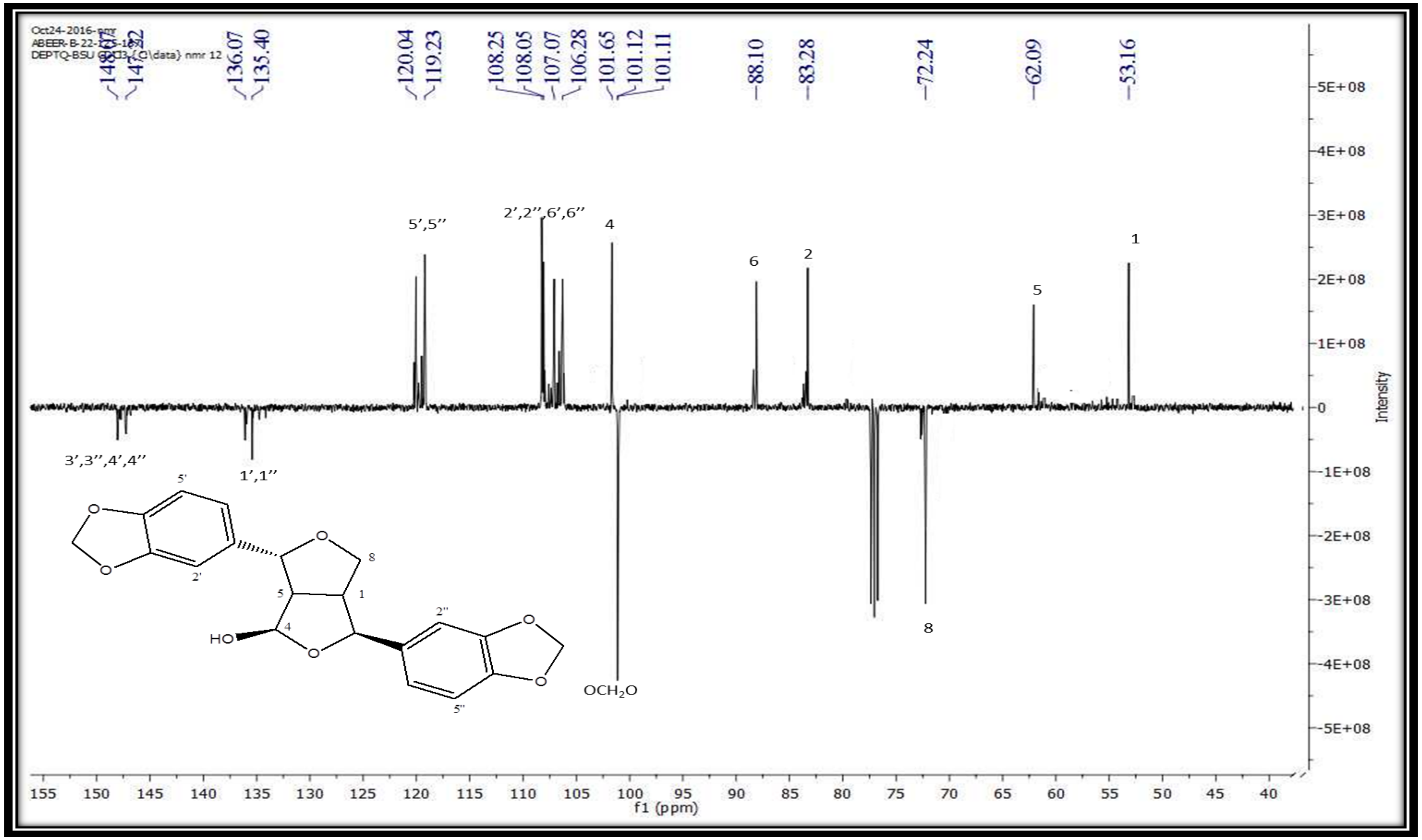

Figure S2. DEPT-Q NMR spectrum of compound **35** measured in $CDCl_3$ at 100 MHz.

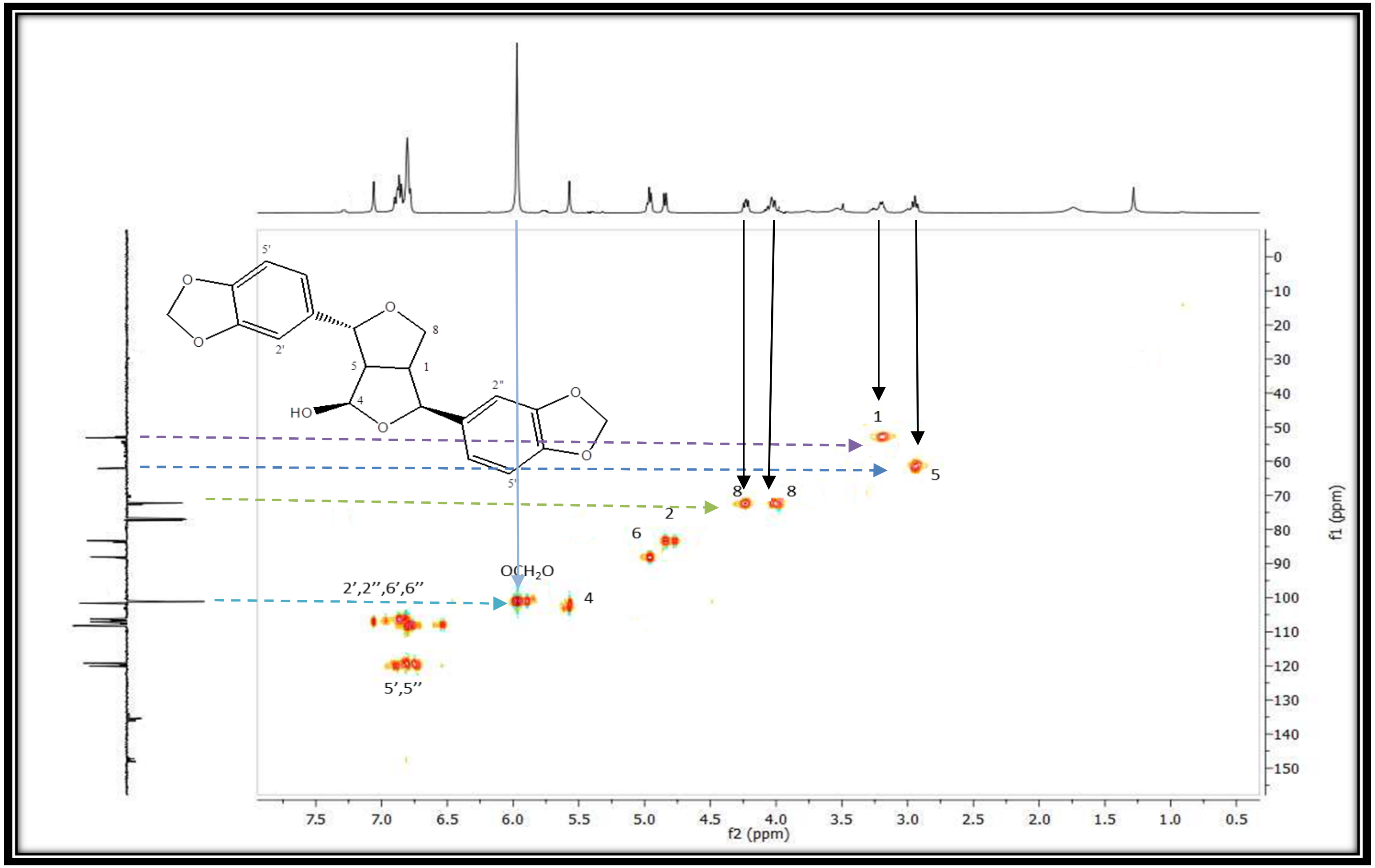

Figure S3. HSQC spectrum of compound **35** measured in $CDCl_3$

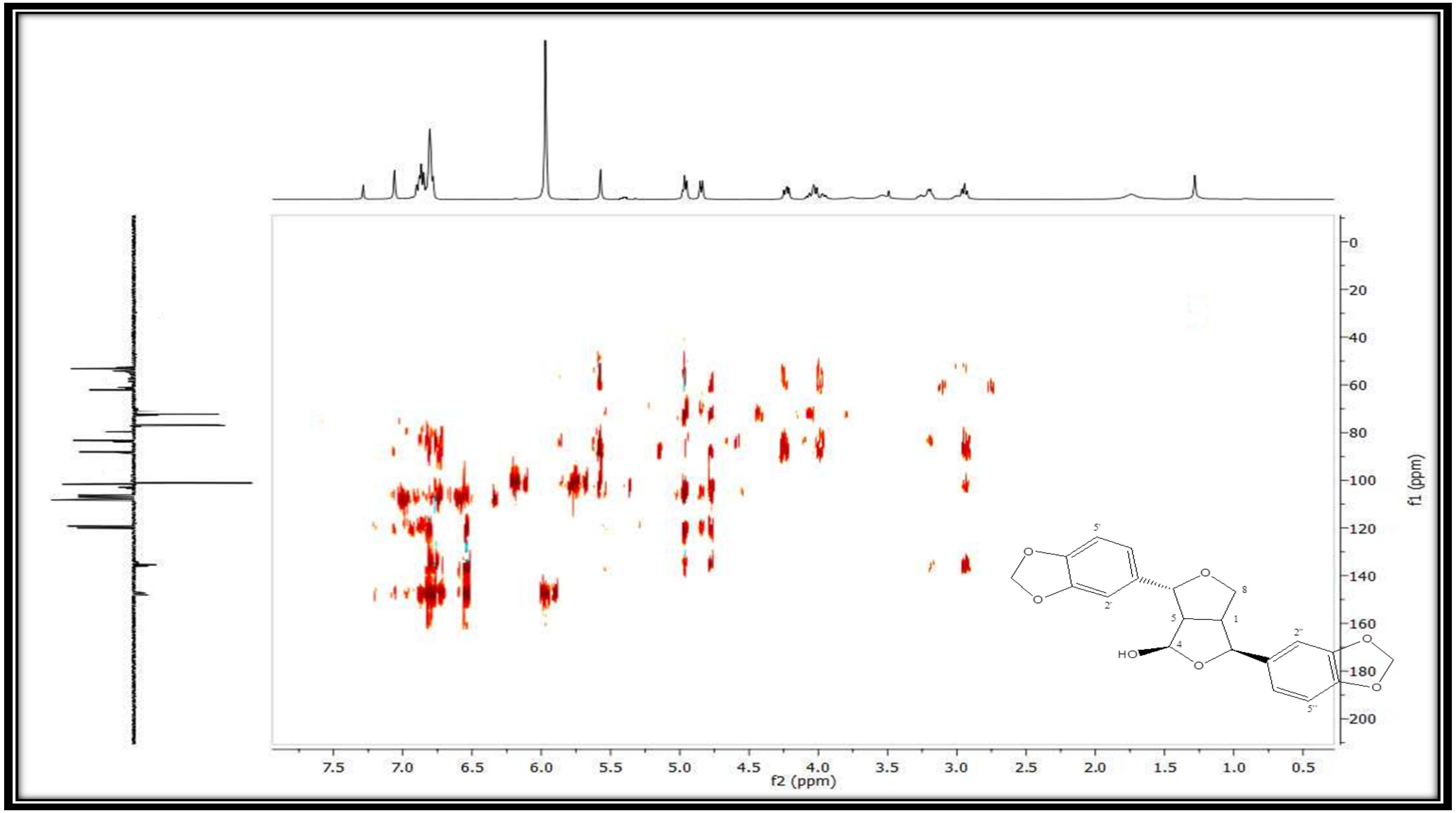

Figure S4. HMBC spectrum of compound **35** measured in $CDCl_3$

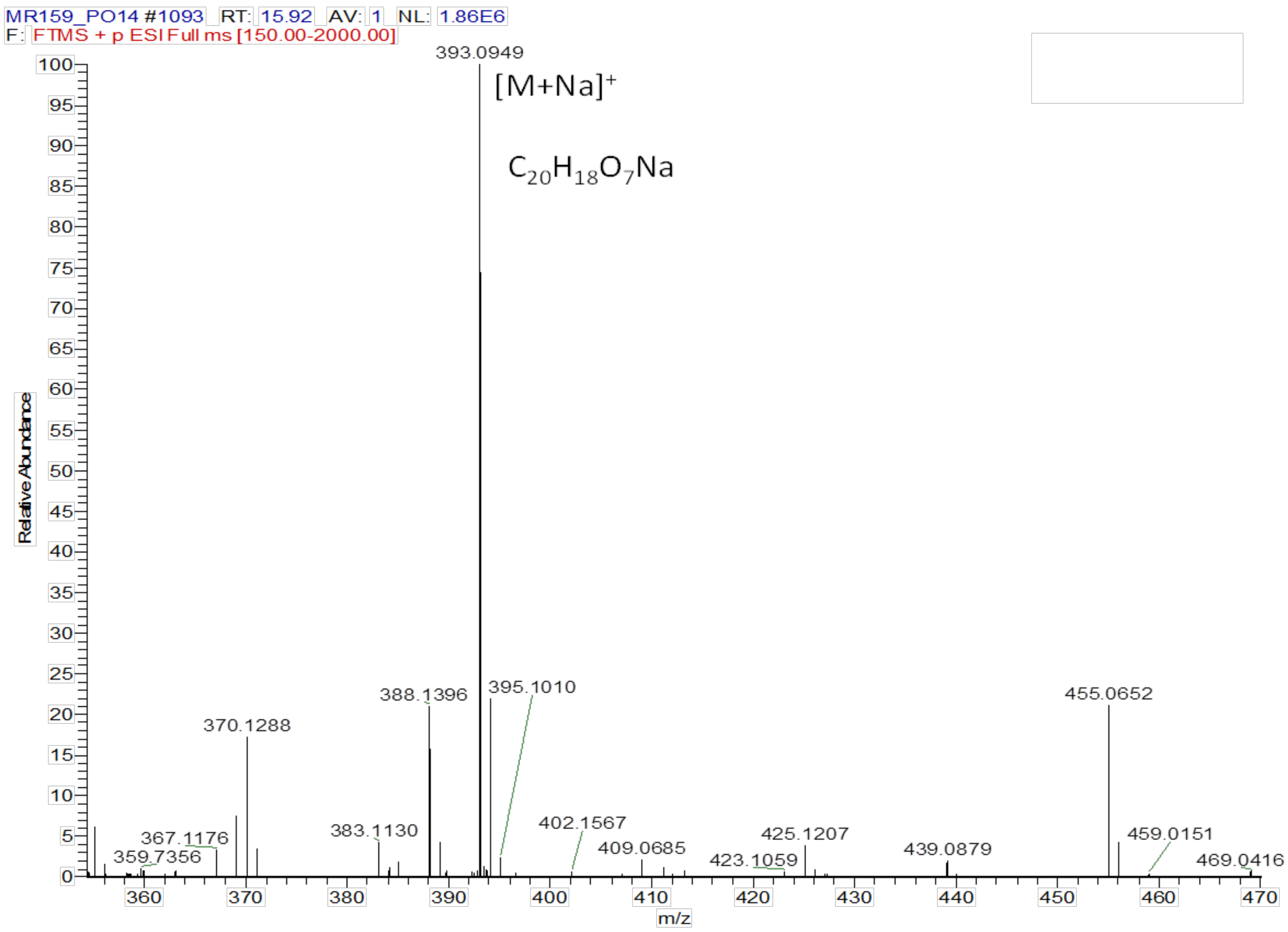

Figure S5. HRESIMS spectrum of compound **35**.
